# Supplementary material for: Antigenic properties of the SARS-CoV-2 nucleoprotein are altered by the RNA admixture
Source: PeerJ. 2022 Jan 7;10:e12751. doi: 10.7717/peerj.12751 (PMC8744485; doi:10.7717/peerj.12751)
Supplement: Supplemental Information 1 [file peerj-10-12751-s001.docx]

**Antigenic properties of the SARS-CoV-2 nucleoprotein are altered by the RNA admixture**

Denis E. Kolesov^1^, Maria V. Sinegubova^1^, Ivan I. Vorobiev^1^, Nadezhda A. Orlova*^1^

**Supporting Figures**

>NP

Msdngpqnqrnapritfggpsdstgsnqngersgarskqrrpqglpnntaswftaltqhgkedlkfprgqgvpintnsspddqigyyrratrrirggdgkmkdlsprwyfyylgtgpeaglpygankdgiiwvategalntpkdhigtrnpannaaivlqlpqgttlpkgfyaegsrggsqassrsssrsrnssrnstpgssrgtsparmagnggdaalalllldrlnqleskmsgkgqqqqgqtvtkksaaeaskkprqkrtatkaynvtqafgrrgpeqtqgnfgdqelirqgtdykhwpqiaqfapsasaffgmsrigmevtpsgtwltytgaiklddkdpnfkdqvillnkhidayktfpptepkkdkkkkadetqalpqrqkkqqtvtllpaadlddfskqlqqsmssadstqahhhhhhhhhh

>NTD

Msdngpqnqrnapritfggpsdstgsnqngersgarskqrrpqglpnntaswftaltqhgkedlkfprgqgvpintnsspddqigyyrratrrirggdgkmkdlsprwyfyylgtgpeaglpygankdgiiwvategalntpkdhigtrnpannaaivlqlpqgttlpkgfyaegsrggsqassrsssrsrnssrnstpgssrgtsparmagnggdaalahhhhhhhhhh

>CTD

Mvlrtsvmllalllldrlnqleskmsgkgqqqqgqtvtkksaaeaskkprqkrtatkaynvtqafgrrgpeqtqgnfgdqelirqgtdykhwpqiaqfapsasaffgmsrigmevtpsgtwltytgaiklddkdpnfkdqvillnkhidayktfpptepkkdkkkkadetqalpqrqkkqqtvtllpaadlddfskqlqqsmssadstqahhhhhhhhhh

**Figure S1. Aminoacid sequences of the nucleocapsid protein (NP) and it’s fragments, as encoded by the plasmids pHYP-NPC-10H, pHYP-NP-head-C-10H, pHYP-NP-tail-C-10H.** Sequences are in FASTA format.

**
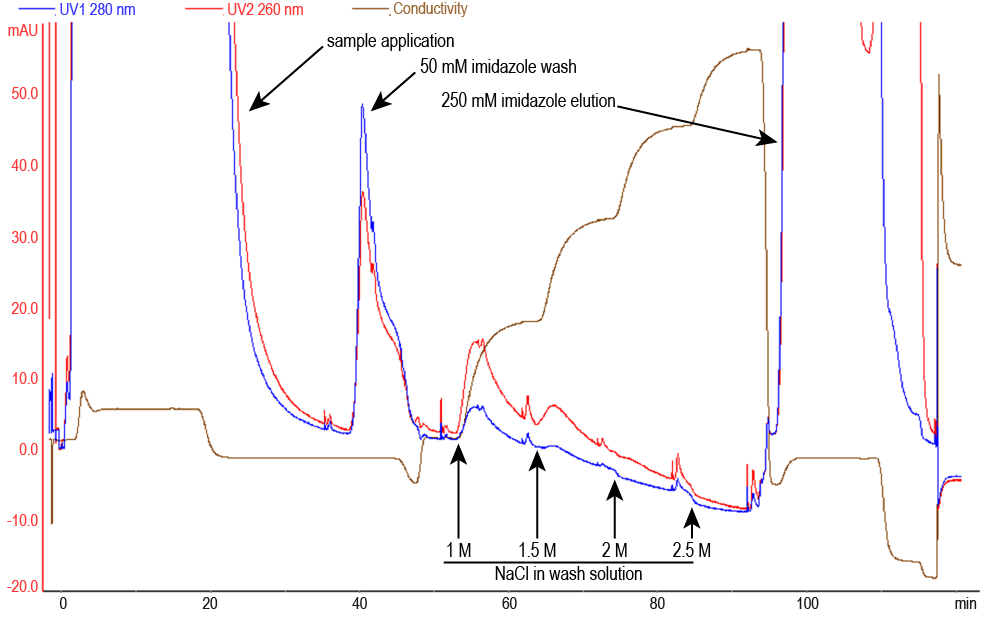
**

**Figure S2. IMAC chromatography trace for the RNase A- treated NP sample, on-column wash by the stepwise NaCl concentration gradient.** Eluate absorbance at 280 nm is in blue, absorbance at 260 nm – in red, conductivity in brown. Ten minutes of the flow stops were performed at the end of each NaCl gradient segment, resulting in appearance of peaks, preceding the eluate from the next segment.


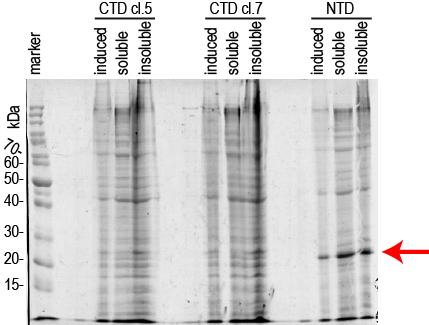


**Figure S3. Absence of the NP CTD expression in the BL21[DE3]/pHYP-NP-CTD strain.** SDS-PAGE analysis of total E.coli proteins, 2 h induction by 1 mM IPTG, 30 oC, total proteins (induced), soluble and insoluble protein fractions. Cl.5, cl. 7 – transformation of the BL21[DE3] cells by two various clones of the pHYP-NP-CTD plasmid.

**
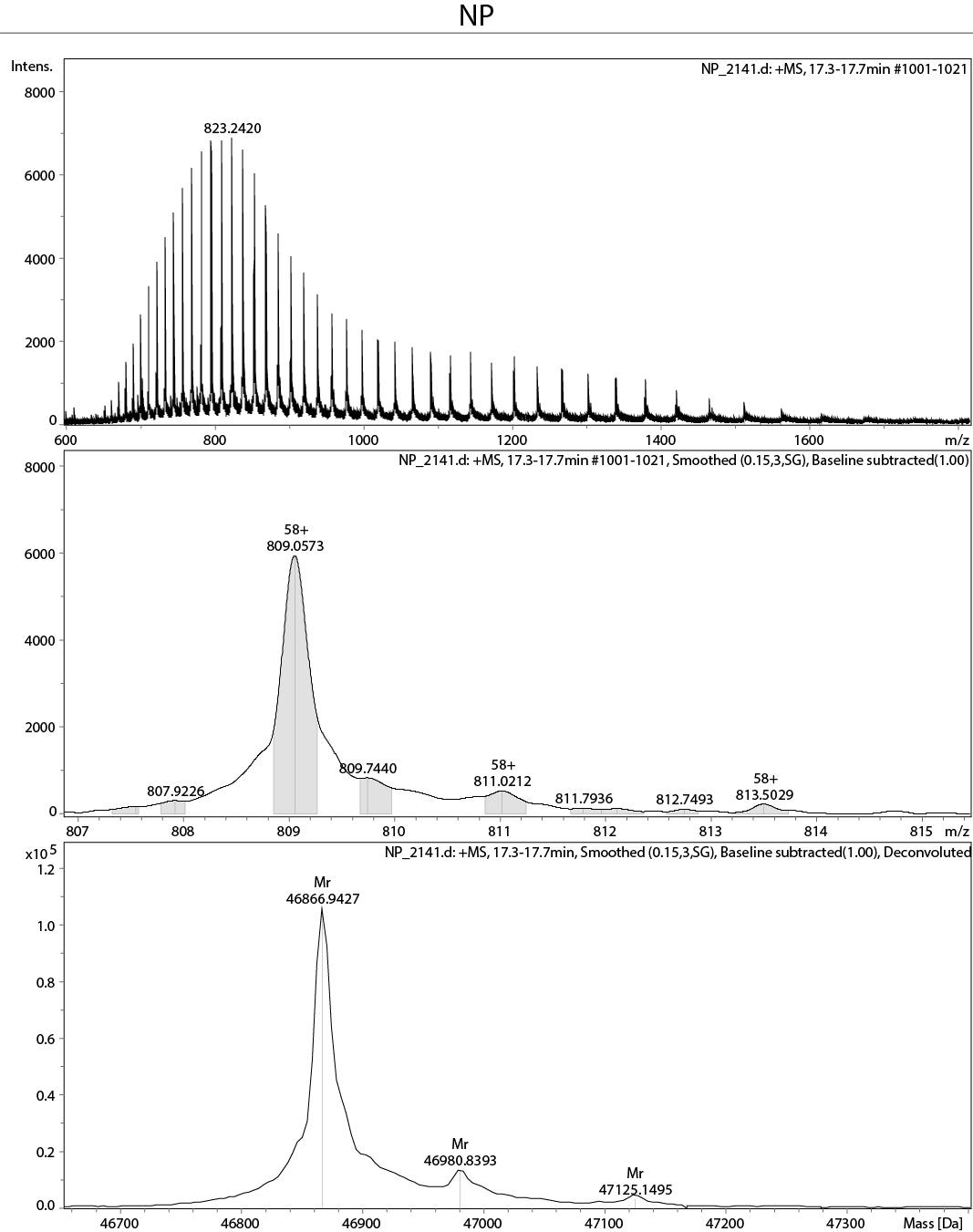
**

**Figure S4. Raw data of the ESI-MS analysis of the full-length NP preparation.**

**
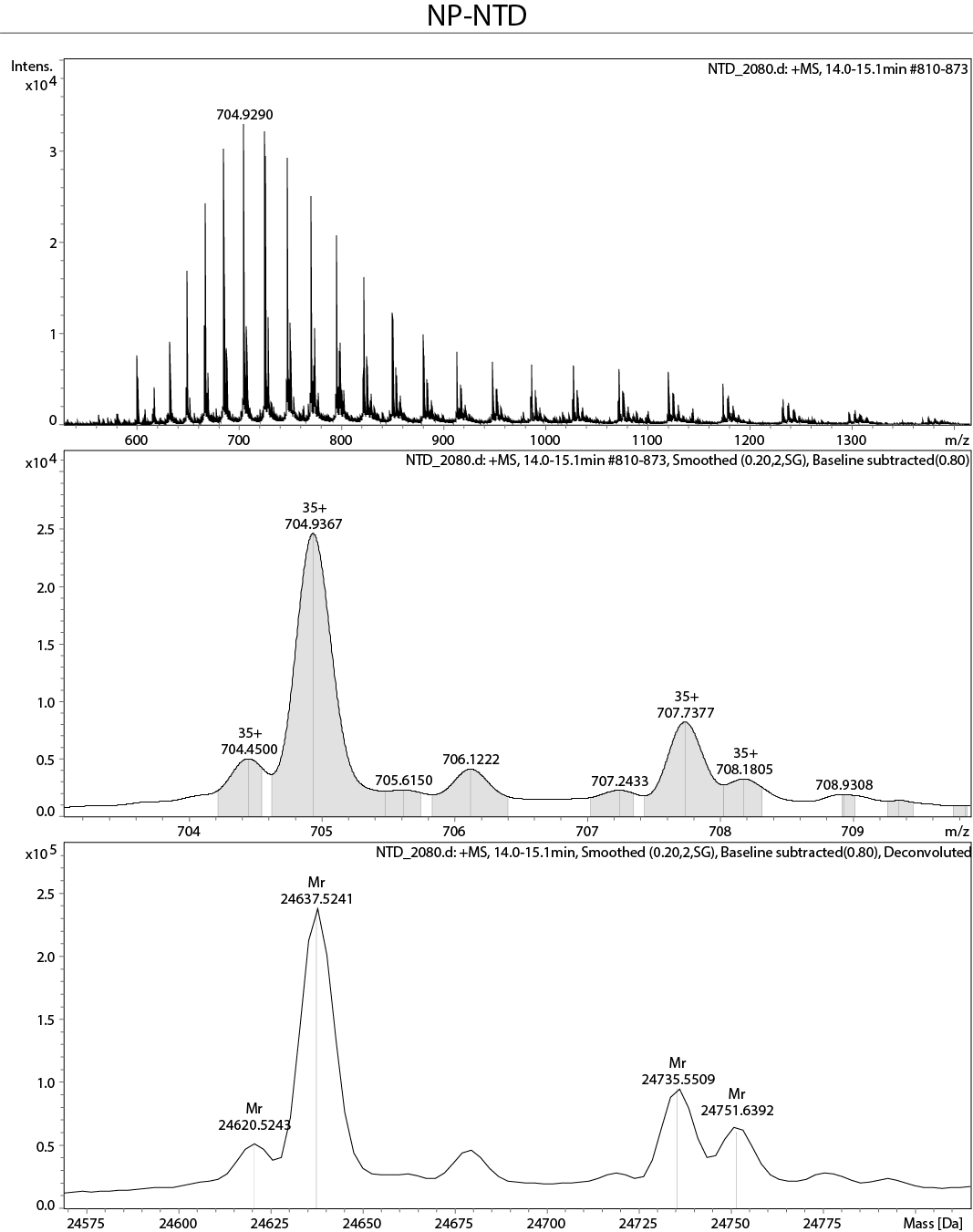
**

**Figure S5. Raw data of the ESI-MS analysis of the NTD preparation.**

**
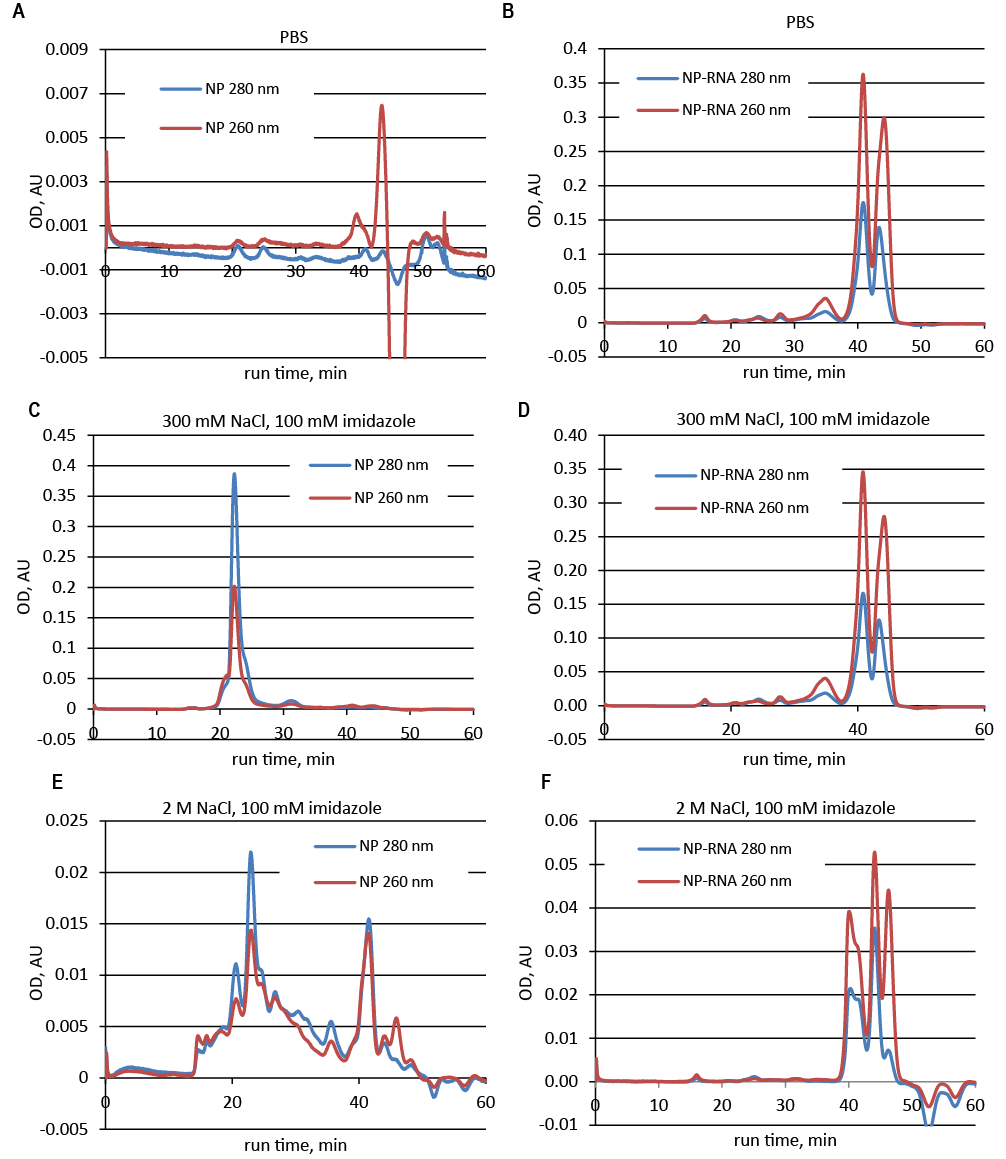
**

**Figure S6. Chromatography traces of the NP and NP-RNA protein preparations size exclusion chromatography analysis, dual wavelength optical detection. (**A), (B) - PBS as the mobile phase; (C), (D) – 300 mM NaCl, 20 mM sodium phosphate pH 7.5, 100 mM imidazole-HCl mobile phase; (E), (F) - 2 M NaCl, 20 mM sodium phosphate pH 7.5, 100 mM imidazole as the mobile phase. Panels (C), (D) are same to the Fig. 2 E, F and are shown here for ease of the direct visual analysis.

**
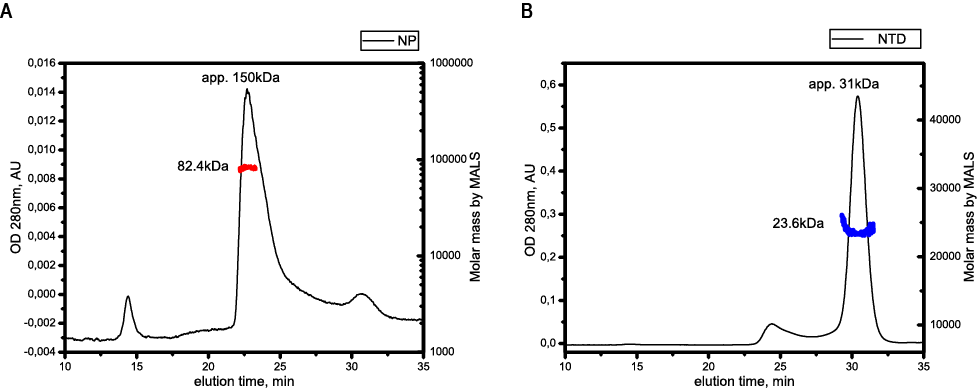
**

**Figure S7. Chromatography traces of the NP and NTD protein preparations size exclusion chromatography analysis, MALS and 280 nm detection**. (A) – NP; (B) – NTD**.** Molar masses, determined by the MALS detector, are shown on left side of peaks, molecular masses, determined by the calibration curve interpolation, are shown above peaks.

**
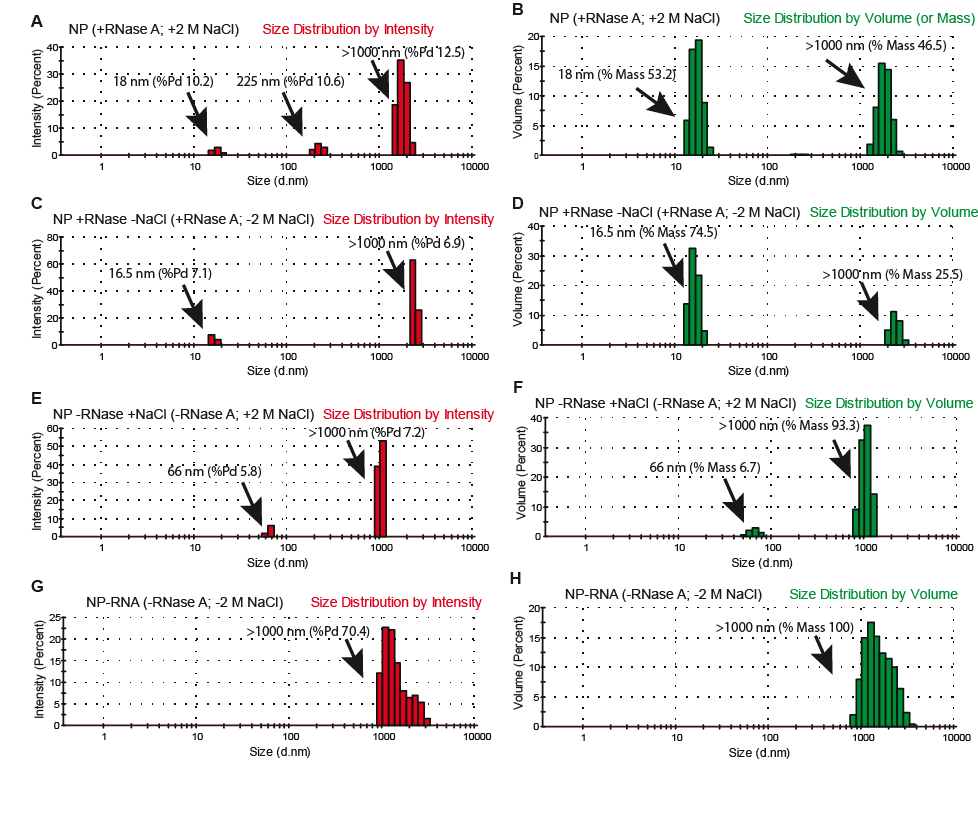
Figure S8. Size distribution by dynamic light scattering** **for NP variants**. (A), (B) – particle size distribution by intensity and volume for the pure NP antigen. (C), (D) – RNase A – treated NP, no 2 M NaCl wash. (E), (F) – Rnase A – untreated NP, with 2 M NaCl wash. (G), (H) – NP-RNA.

**
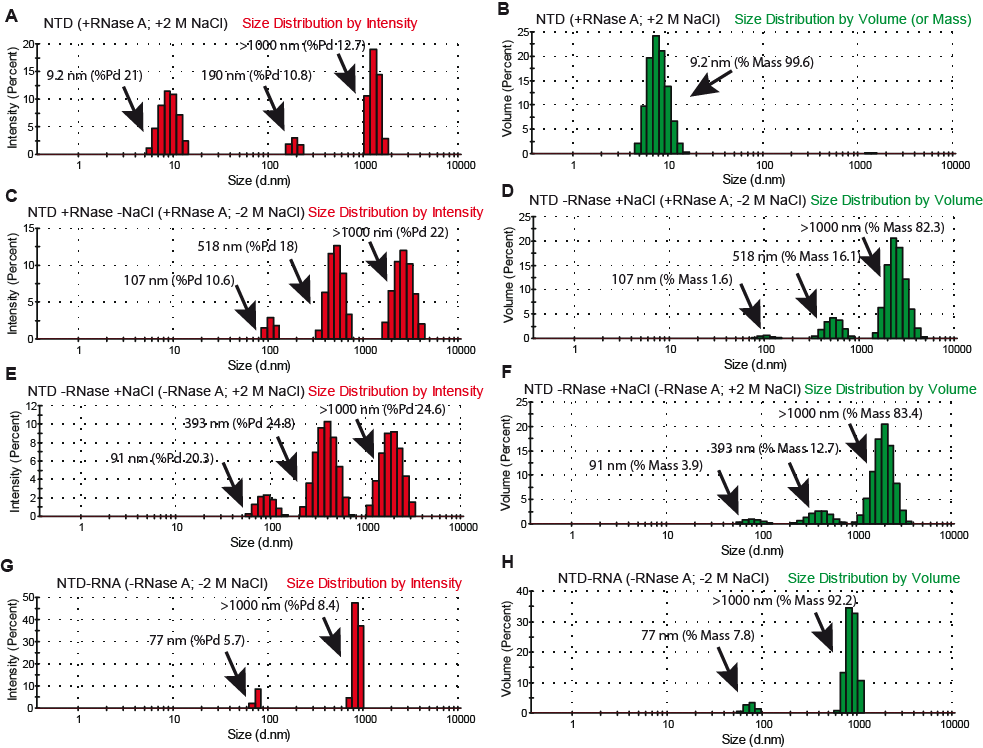
**

**Figure S9. Size distribution by dynamic light scattering** **for NTD variants**. (A), (B) – particle size distribution by intensity and volume for the pure NTD antigen. (C), (D) – RNase A – treated NTD, no 2 M NaCl wash. (E), (F) – Rnase A – untreated NTD, with 2 M NaCl wash. (G), (H) – NTD-RNA.

**
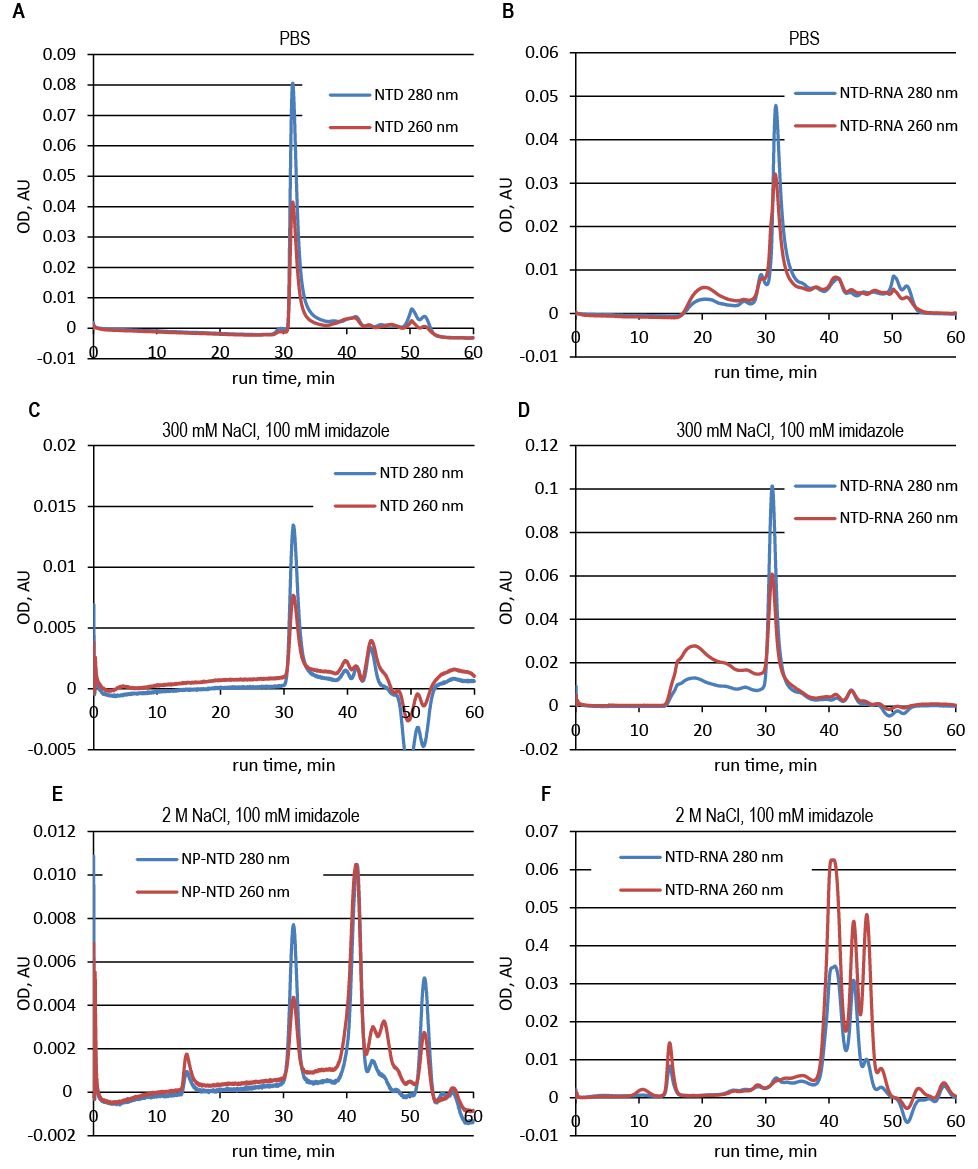
**

**Figure S10. Chromatography traces of the NTD and NTD-RNA protein preparations size exclusion chromatography analysis, dual wavelength optical detection. (**A), (B) - PBS as the mobile phase; (C), (D) – 300 mM NaCl, 20 mM sodium phosphate pH 7.5, 100 mM imidazole-HCl mobile phase; (E), (F) - 2 M NaCl, 20 mM sodium phosphate pH 7.5, 100 mM imidazole as the mobile phase.

**
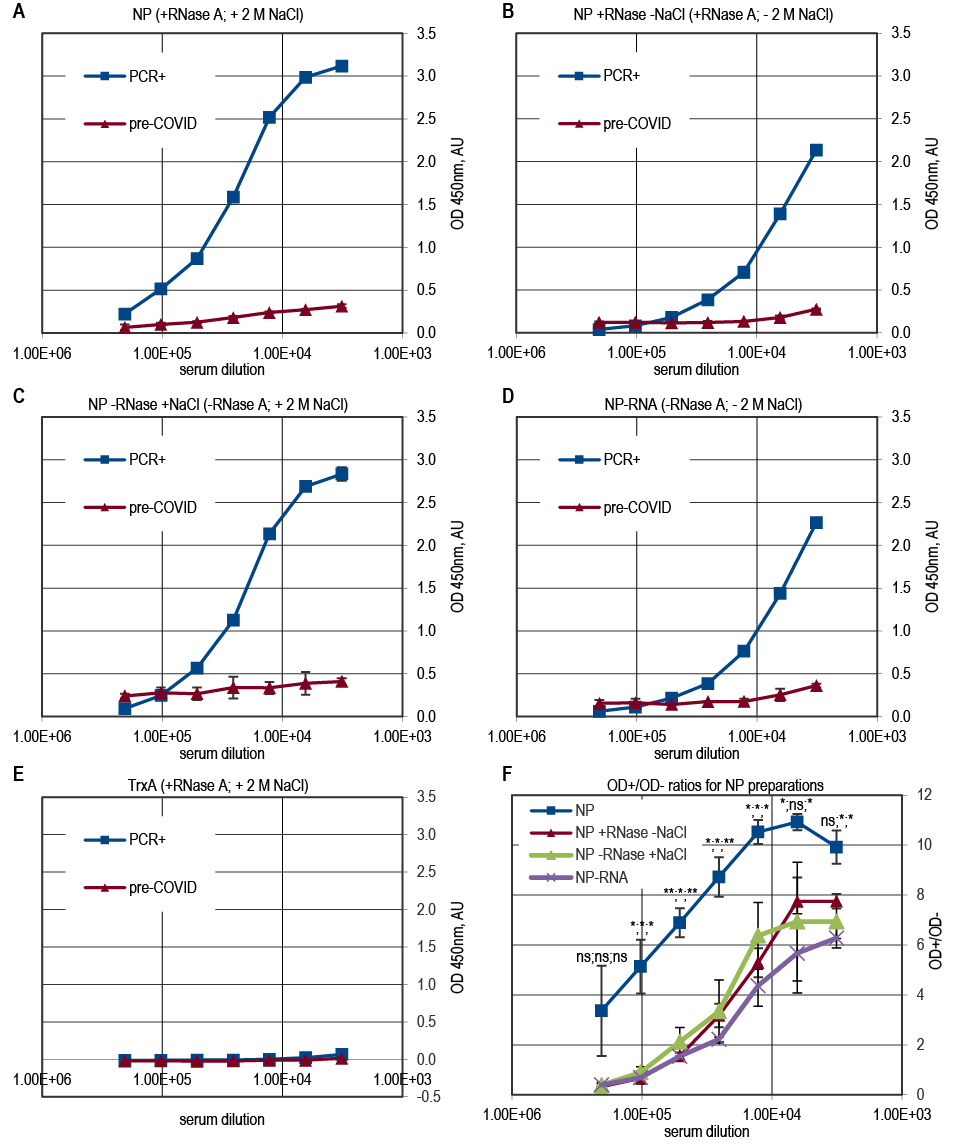
**

**Figure S11. Antibody capture ELISA and dynamics of the assay specificity ratios for various purification methods, NP antigen**. (A) – pure NP antigen, protein preparation and the ELISA test was independent from the test shown on Figure 3. (B) – NP protein antigen, treated by the RNase A and not treated by the on-column 2 M NaCl wash. (C) - NP protein antigen, not treated by the RNase A and treated by the on-column 2 M NaCl wash. (D) – NP-RNA antigen, protein preparation and the ELISA test was independent from the test shown on Figure 3. E – negative control TrxA antigen, purified exactly as the NP antigen. (F) – (OD+/OD-) for various serum samples dilutions, calculated as the ratios of OD readings for the PCR+ sample and the pre-COVID sample for the same sample dilutions. Blue lines - pooled PCR+ sera. Red lines - pooled pre-COVID sera. Statistical analysis by the or one-way ANOVA with the post-hoc Tukey-Kramer HSD test, n=2, * - p < 0.05; ** - p < 0.01; *** - p < 0.001, ns – non-significant. P-values are presented for the pure NP antigen versus three other antigen preparations as “NP vs. NP+-; NP vs. NP-+; NP vs. NP-RNA”.

**
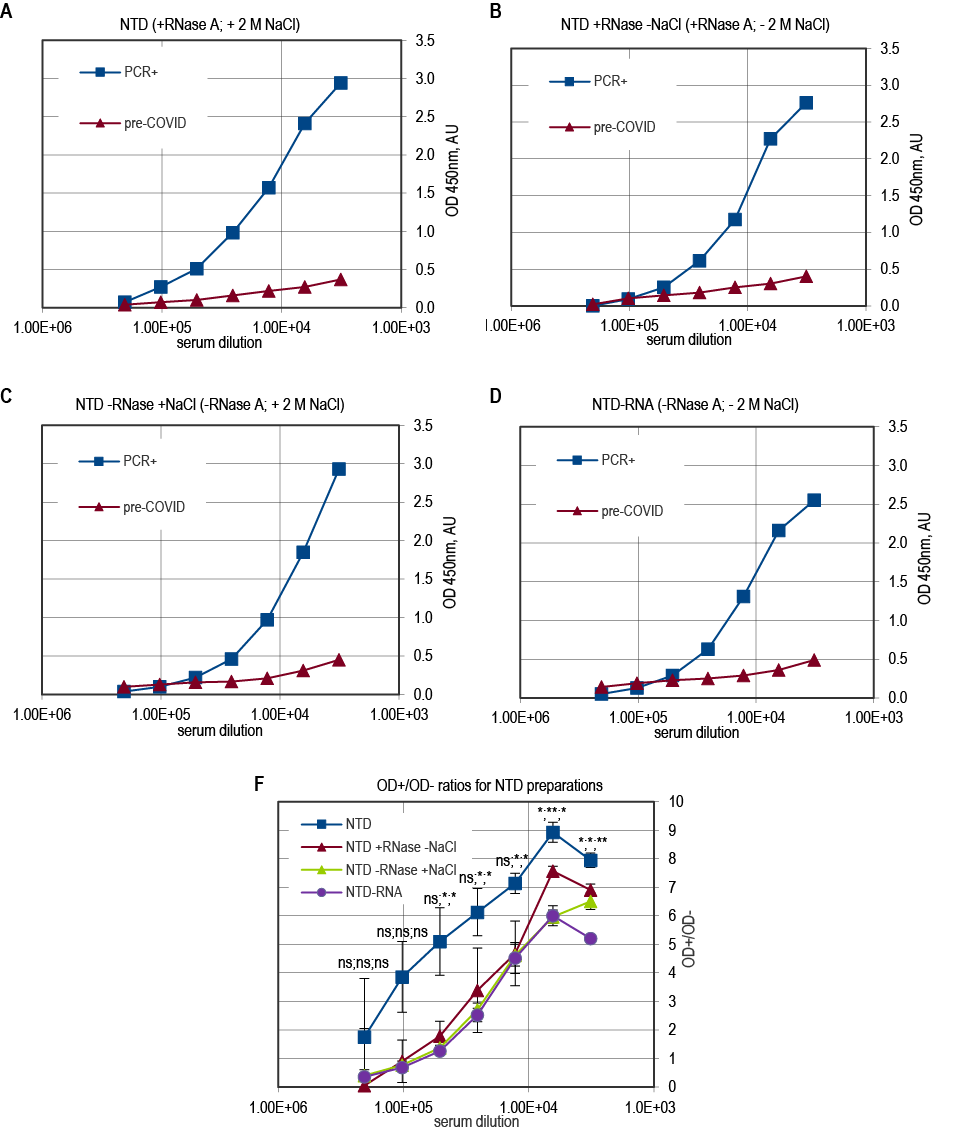
**

**Figure S12. Antibody capture ELISA and dynamics of the assay specificity ratios for various purification methods, NTD antigen**. (A) – pure NTD antigen, protein preparation and the ELISA test was independent from the test shown on Figure 3. (B) – NTD protein antigen, treated by the RNase A and not treated by the on-column 2 M NaCl wash. (C) - NTD protein antigen, not treated by the RNase A and treated by the on-column 2 M NaCl wash. (D) – NTD-RNA antigen. (E) – test specificity ratios (OD+/OD-) for various serum samples dilutions, calculated as the ratios of OD readings for the PCR+ sample and the pre-COVID sample at the same sample dilutions. Blue lines - pooled PCR+ sera. Red lines - pooled pre-COVID sera. Statistical analysis by the or one-way ANOVA with the post-hoc Tukey-Kramer HSD test, n=2, * - p < 0.05; ** - p < 0.01; *** - p < 0.001, ns – non-significant. P-values are presented for the pure NP antigen versus three other antigen preparations as “NTD vs. NTD+-; NTD vs. NTD-+; NTD vs. NTD-RNA”.

**Supporting Table 1. Description of control tests, used for correlation analysis.**

| **Name** | **Antigen** | **Quantification method *** | **Test description** | **Code in Fig. 4** |
| --- | --- | --- | --- | --- |
| EUROIMMUN Anti-SARS-CoV2 ELISA IgG assay | S1 | OD/CO | <https://www.fda.gov/media/137609/download> | S1 |
| Siemens SARS-CoV-2 IgG assay | RBD | S/CO | https://www.siemens-healthineers.com/en-us/laboratory-diagnostics/assays-by-diseases-conditions/infectious-disease-assays/sars-cov-2-igg-assay | RBD |
| DiaPro COVID-19 IgG assay | S1/S2 | S/CO | https://www.launchdiagnostics.com/product-list/dia-pro-covid-19-spike-1-and-2-igg-elisa/ | S1/S2 |
| EUROIMMUN Anti- SARS-CoV-2 NCP ELISA | NP | OD/CO | https://www.coronavirus-diagnostics.com/documents/Indications/Infections/Coronavirus/EI_2606_D_UK_C.pdf | NP #1 |
| PHE Colindale Anti- SARS-CoV-2 ELISA in-house | NP | S/CO | --- | NP #2 |

* S/CO – signal to cut-off ratio; OD/CO – sample optical density to cut-off optical density ratio quantification methods.
