# Supplementary material for: Antigenic properties of the SARS-CoV-2 nucleoprotein are altered by the RNA admixture
Source: PeerJ. 2022 Jan 7;10:e12751. doi: 10.7717/peerj.12751 (PMC8744485; doi:10.7717/peerj.12751)
Supplement: Supplemental Information 2 — Sequences are in FASTA format. [file peerj-10-12751-s002.pdf]

>NP

MSDNGPQNQRNAPRITFGGPSDSTGSNQNGERSGARSKQRRPQGLPNNTASWFTALTQHGKEDLKFPRGQGVPINT
NSSPDDQIGYYRRATRRIRGGDGKMKDLSPRWYFYYLGTGPEAGLPYGANKDGIIWVATEGALNTPKDHIGTRNPAN
NAAIVLQLPQGTTLPKGFYAEGSRGGSQASSRSSSRSRNSSRNSTPGSSRGTSPARMAGNGGDAALALLLLDRLNQLES
KMSGKGQQQQGQTVTKKSAAEASKKPRQKRTATKAYNVTQAFGRRGPEQTQGNFGDQELIRQGTDYKHWPQIAQF
APSASAFFGMSRIGMEVTPSGTWLTYTGAIKLDDKDPNFKDQVILLNKHIDAYKTFPPTEPKKDKKKADETQALPQRQ
KKQQTVTLLPAADLDDFSKQLQQSMSSADSTQAHHHHHHHHHH

>NTD

MSDNGPQNQRNAPRITFGGPSDSTGSNQNGERSGARSKQRRPQGLPNNTASWFTALTQHGKEDLKFPRGQGVPINT
NSSPDDQIGYYRRATRRIRGGDGKMKDLSPRWYFYYLGTGPEAGLPYGANKDGIIWVATEGALNTPKDHIGTRNPAN
NAAIVLQLPQGTTLPKGFYAEGSRGGSQASSRSSSRSRNSSRNSTPGSSRGTSPARMAGNGGDAALAHHHHHHHHHH

>CTD

MVLRTSVMLLALLLLDRLNQLESKMSGKGQQQQGQTVTKKSAAEASKKPRQKRTATKAYNVTQAFGRRGPEQTQGN
FGDQELIRQGTDYKHWPQIAQFAPSASAFFGMSRIGMEVTPSGTWLTYTGAIKLDDKDPNFKDQVILLNKHIDAYKTFP
PTEPKKDKKKADETQALPQRQKKQQTVTLLPAADLDDFSKQLQQSMSSADSTQAHHHHHHHHHH
