# Supplementary figures and images for: Antigenic properties of the SARS-CoV-2 nucleoprotein are altered by the RNA admixture
Source: PeerJ. 2022 Jan 7;10:e12751. doi: 10.7717/peerj.12751 (PMC8744485; doi:10.7717/peerj.12751)

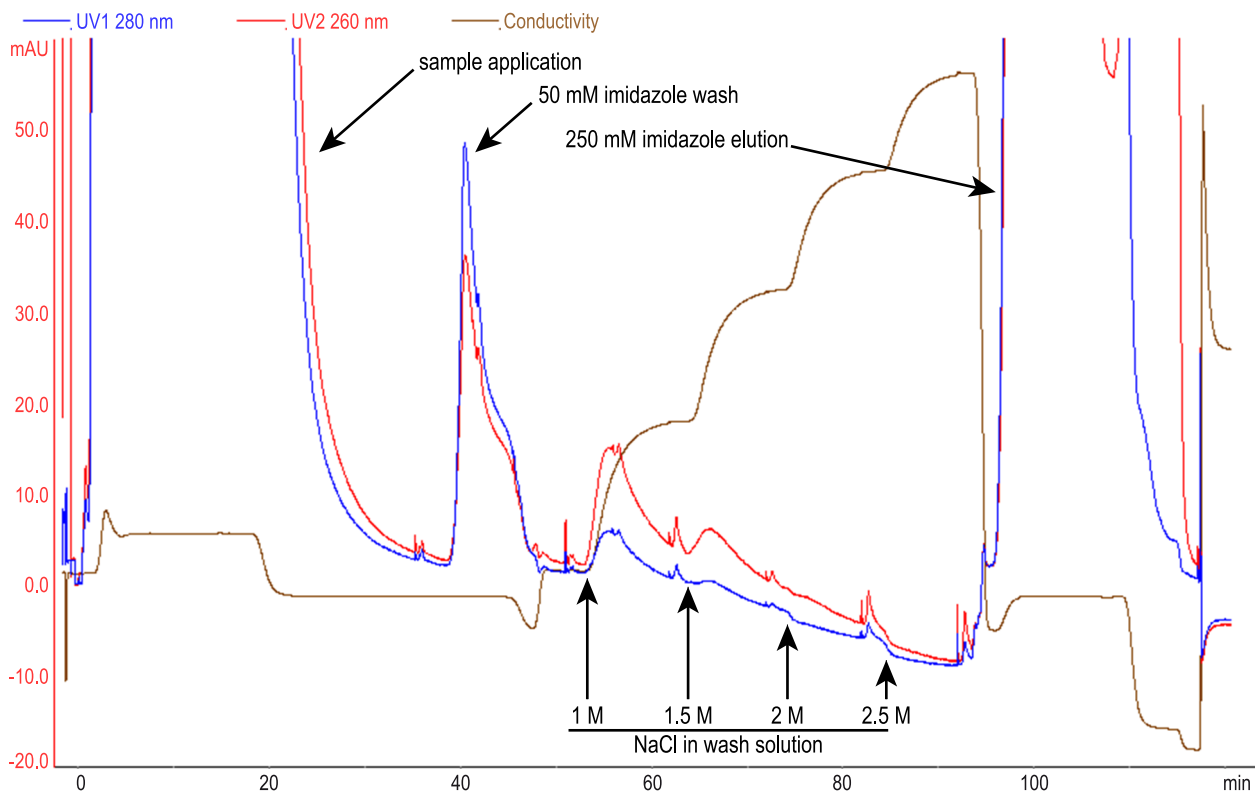

Supplement: Supplemental Information 3 — Eluate absorbance at 280 nm is in blue, absorbance at 260 nm –in red, conductivity in brown. Ten minutes of the flow stops were performed at the end of each NaCl gradient segment, resulting in appearance of peaks, preceding the eluate from the next segment. [file peerj-10-12751-s003.pdf]

# NP

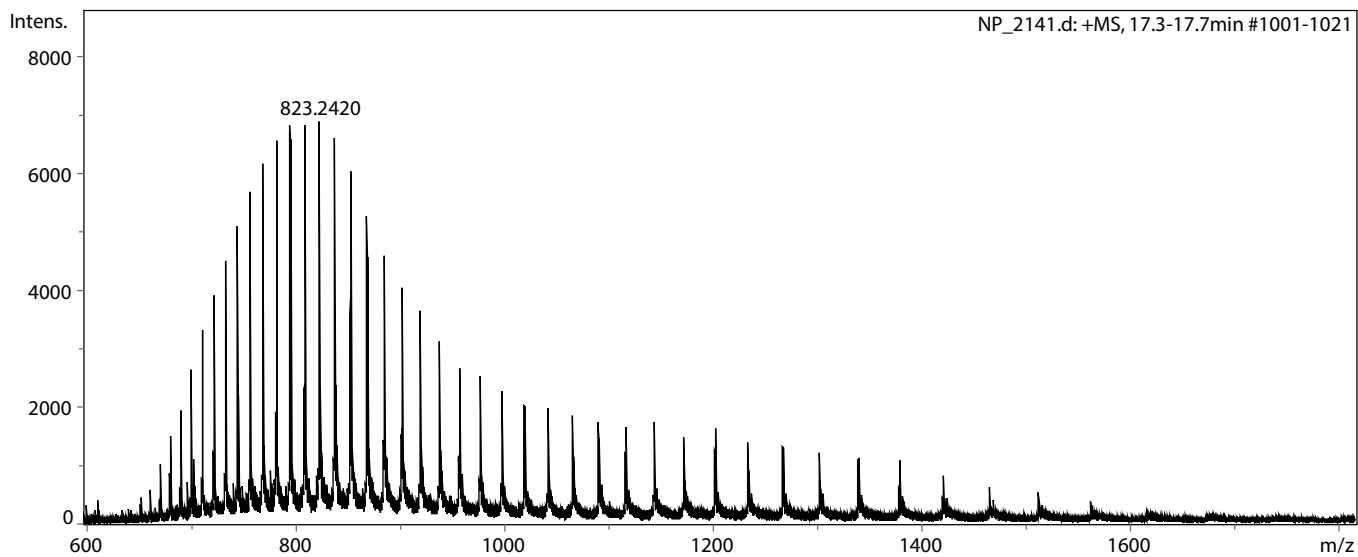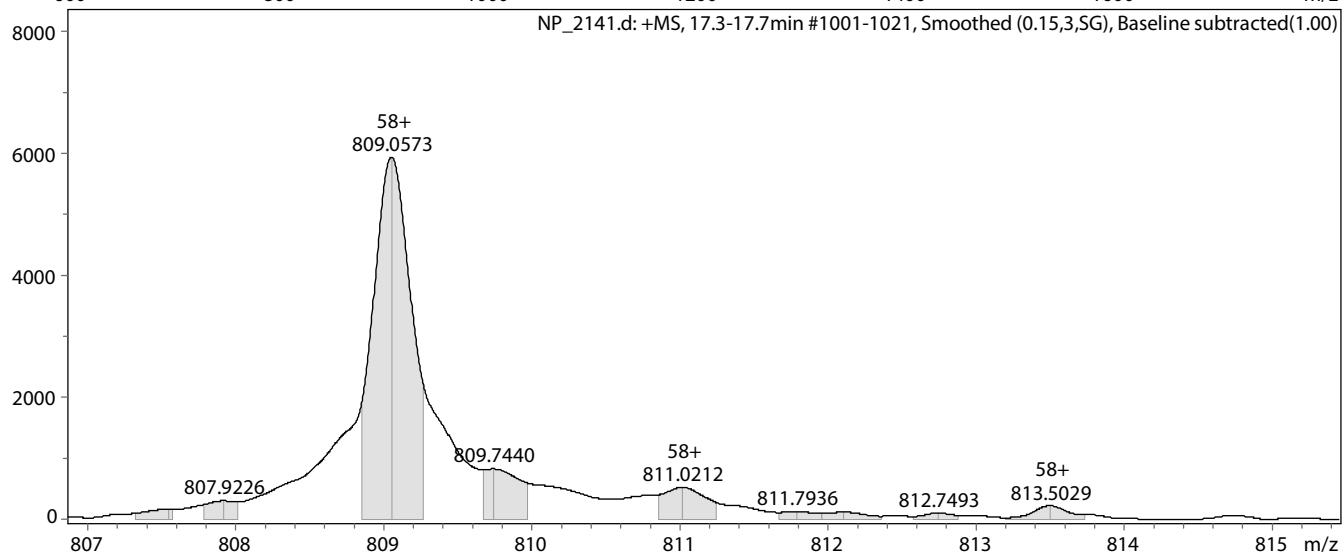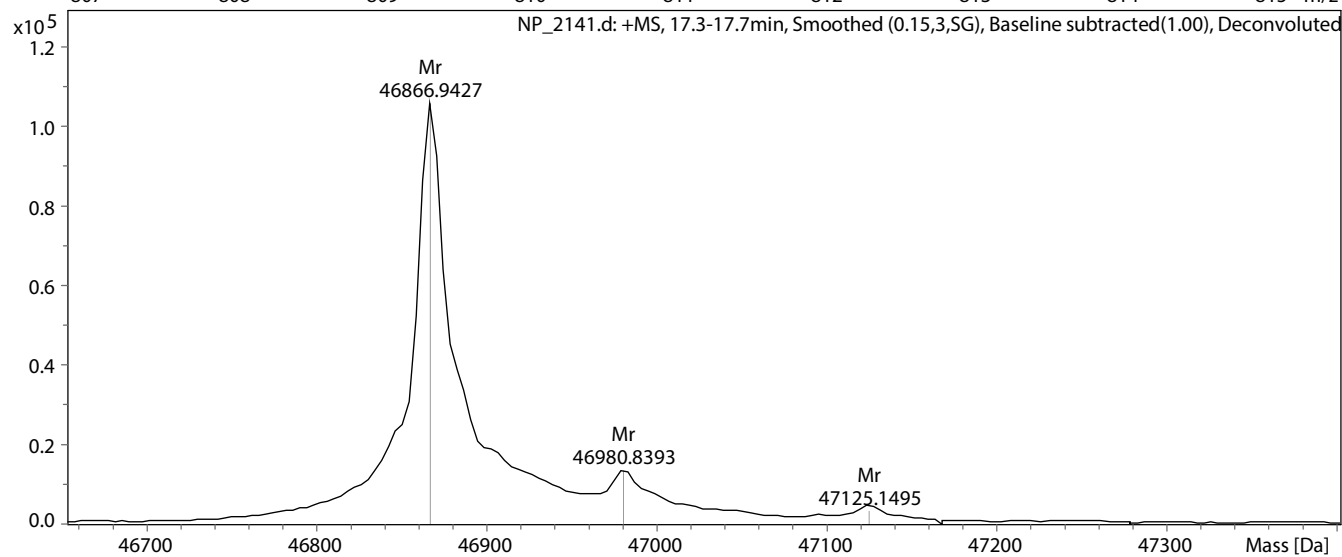

Supplement: Supplemental Information 5 [file peerj-10-12751-s005.pdf]

# NP-NTD

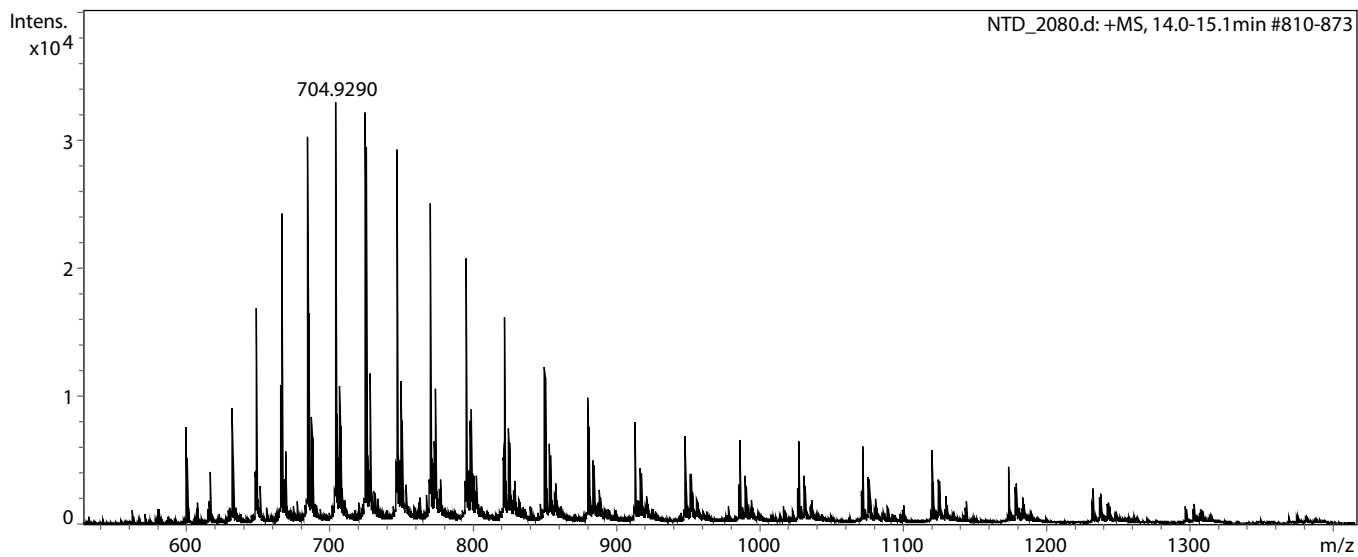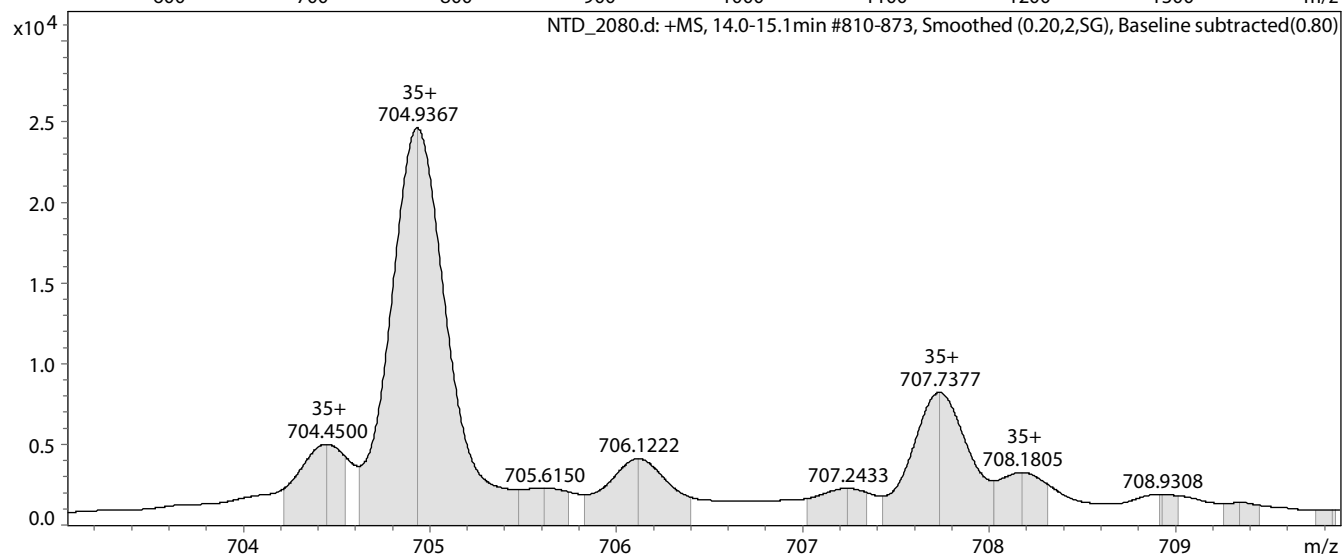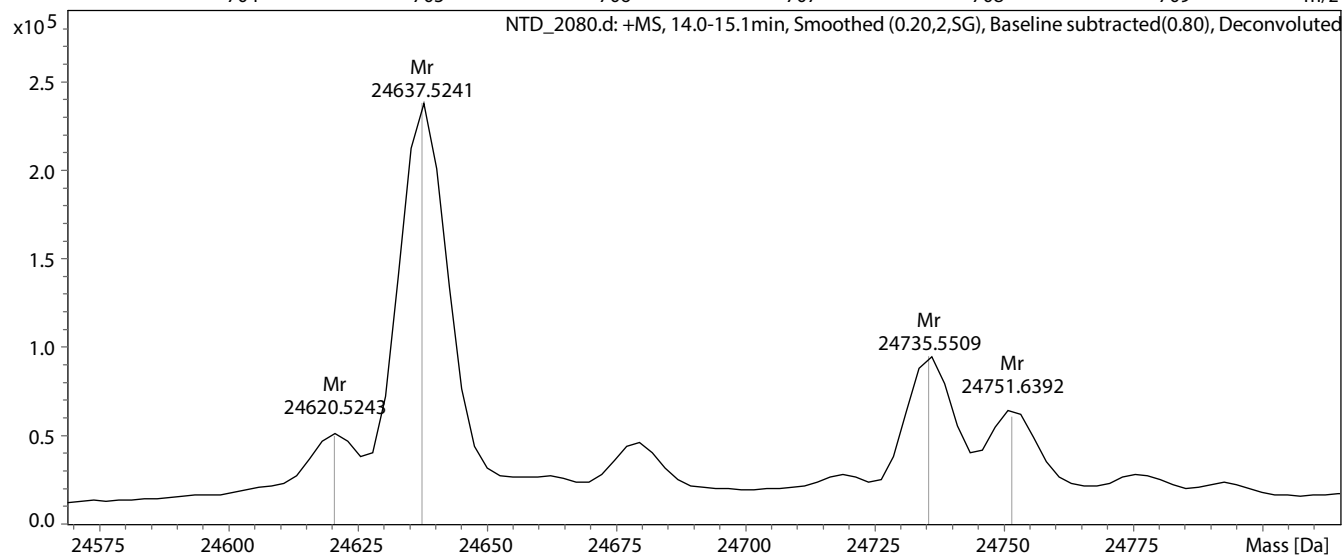

Supplement: Supplemental Information 6 [file peerj-10-12751-s006.pdf]

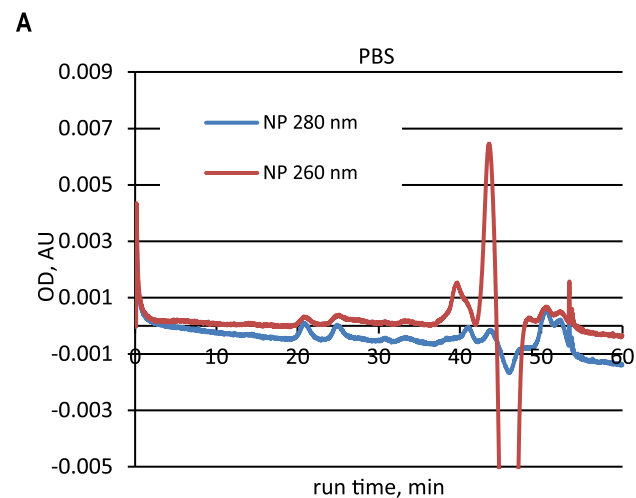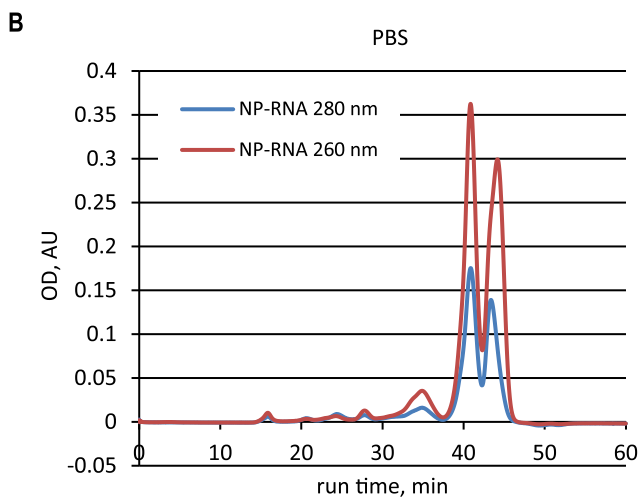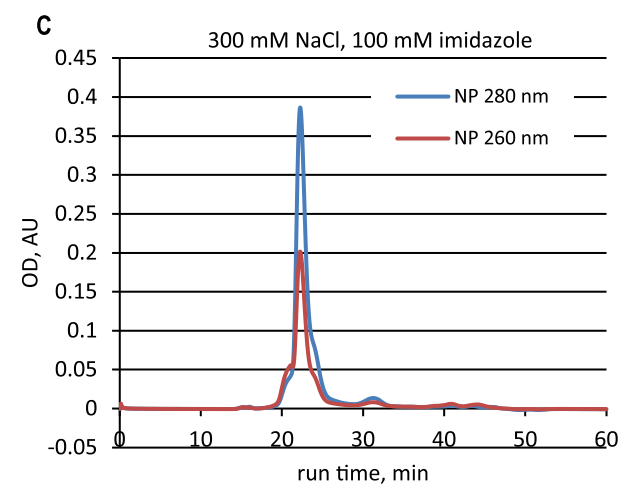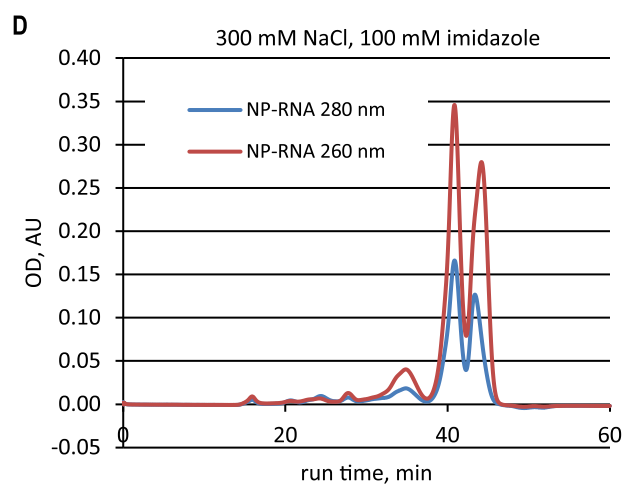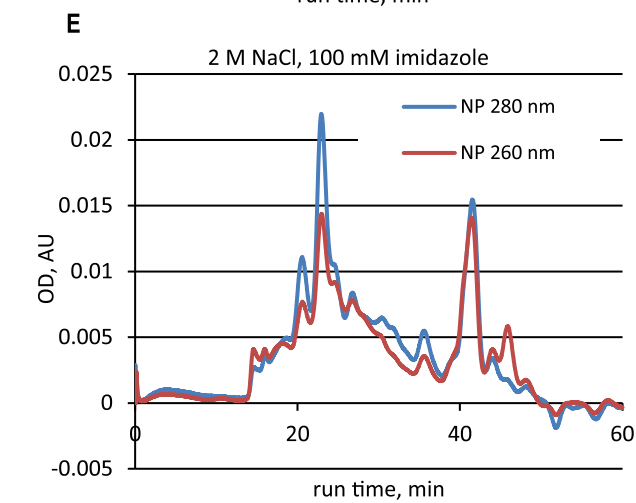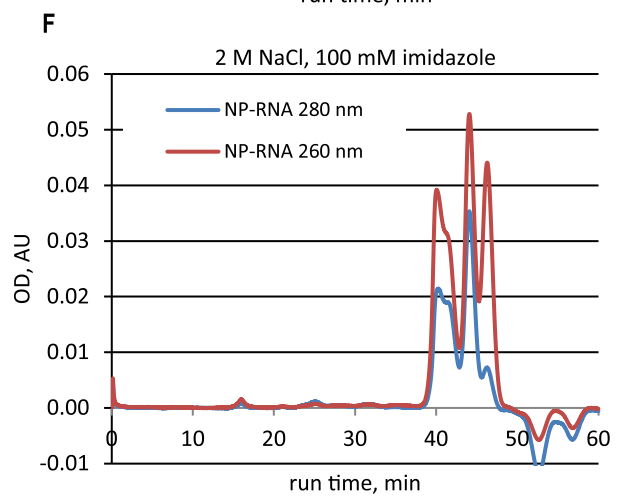

Supplement: Supplemental Information 7 — (A, B) PBS as the mobile phase; (C, D) 300 mM NaCl, 20 mM sodium phosphate pH 7.5, 100 mM imidazole-HCl mobile phase; (E, F) 2 M NaCl, 20 mM sodium phosphate pH 7.5, 100 mM imidazole as the mobile phase. (C, D) are same to the Figs. 2E 2F and are shown here for ease of the direct visual analysis. [file peerj-10-12751-s007.pdf]

**A**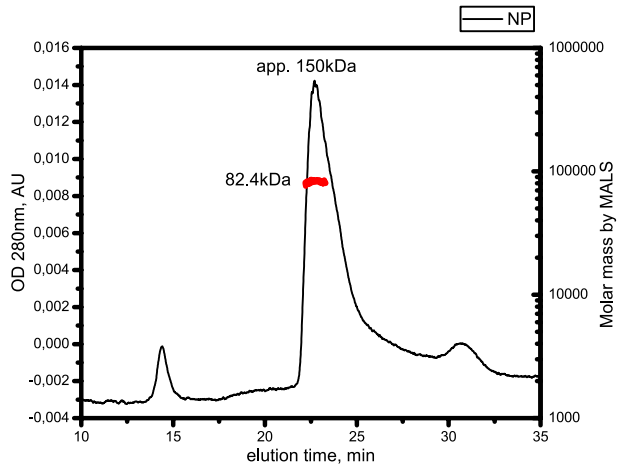**B**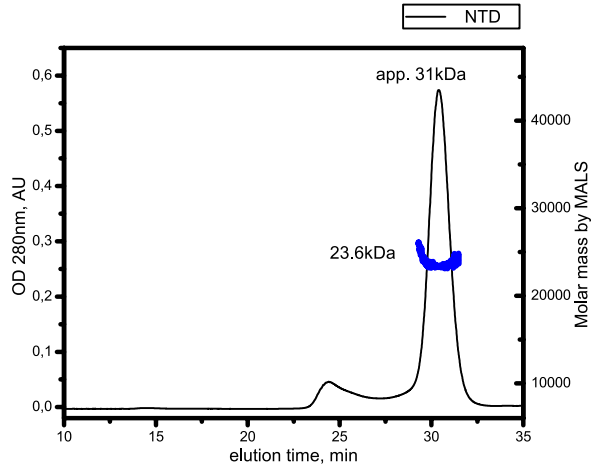

Supplement: Supplemental Information 8 — (A) NP; (B) NTD. Molar masses, determined by the MALS detector, are shown on left side of peaks, molecular masses, determined by the calibration curve interpolation, are shown above peaks. [file peerj-10-12751-s008.pdf]

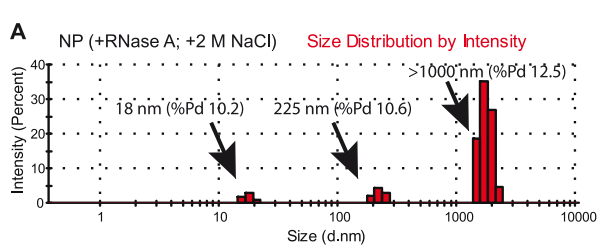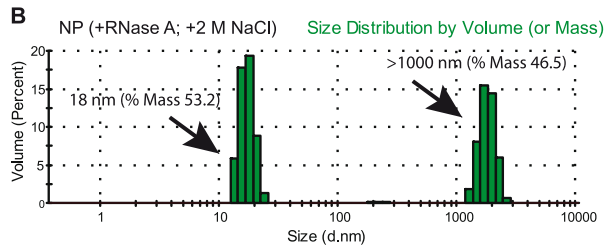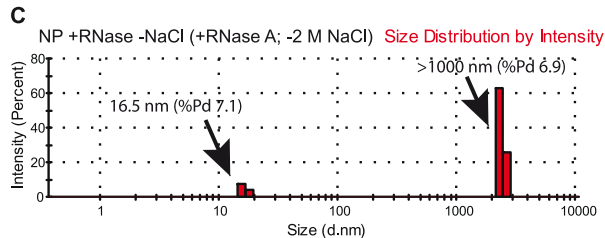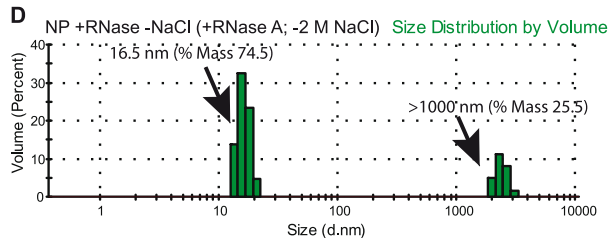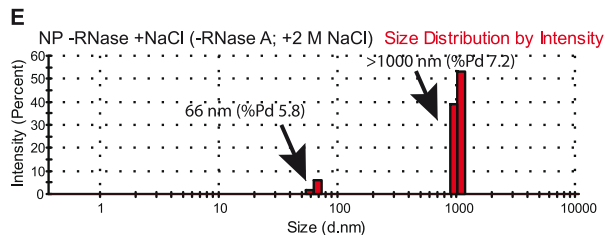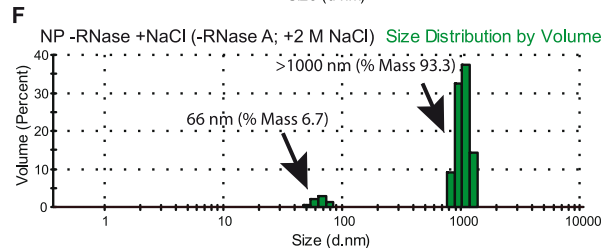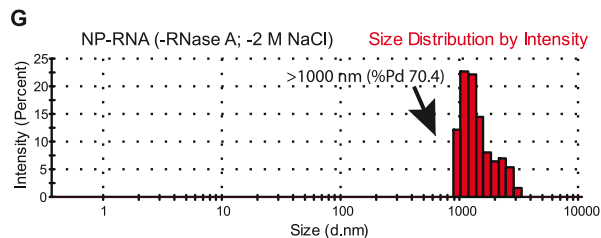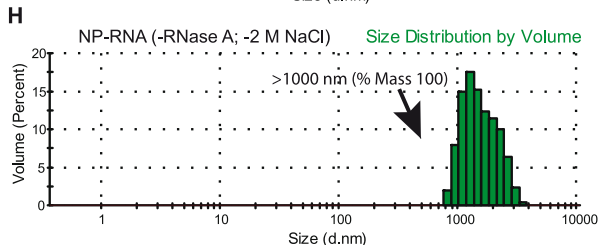

Supplement: Supplemental Information 9 — (A, B) particle size distribution by intensity and volume for the pure NP antigen. (C, D) RNase A–treated NP, no 2 M NaCl wash. (E, F) Rnase A–untreated NP, with 2 M NaCl wash. (G, H) NP-RNA. [file peerj-10-12751-s009.pdf]

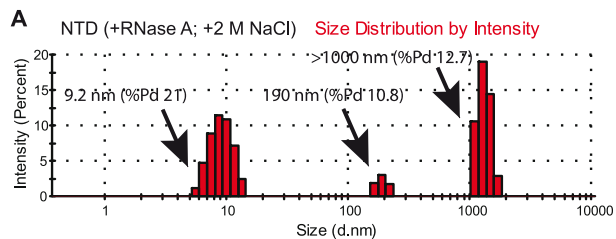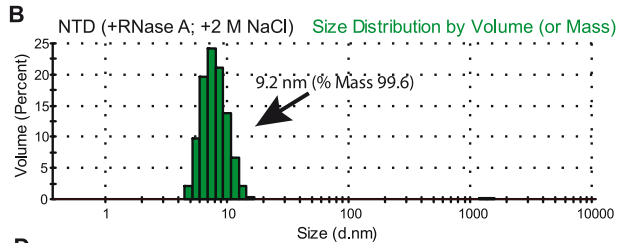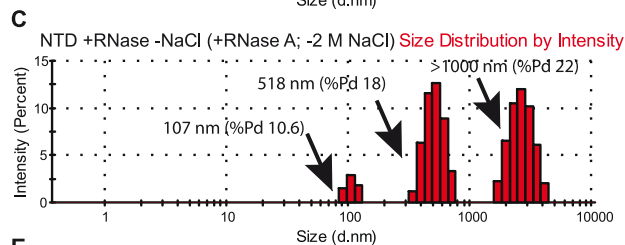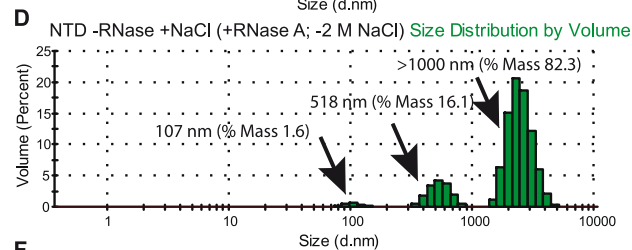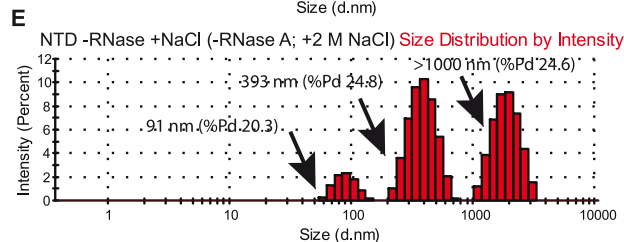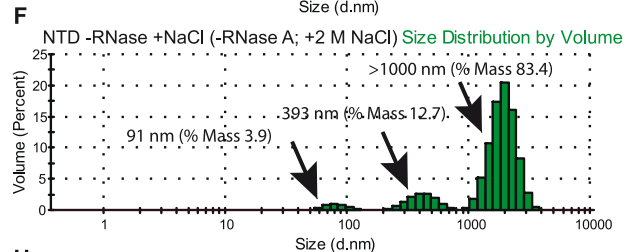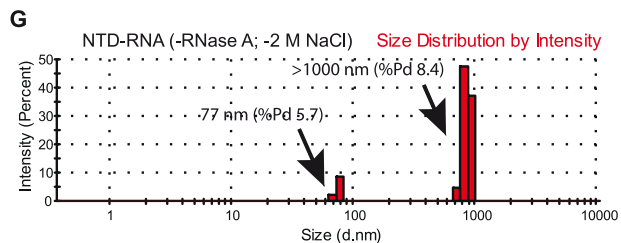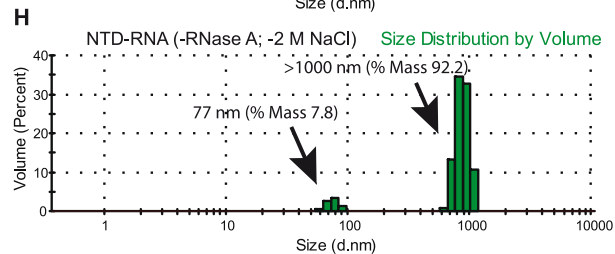

Supplement: Supplemental Information 10 — (A, B) particle size distribution by intensity and volume for the pure NTD antigen. (C, D) RNase A–treated NTD, no 2 M NaCl wash. (E, F) Rnase A–untreated NTD, with 2 M NaCl wash. (G, H) NTD-RNA. [file peerj-10-12751-s010.pdf]

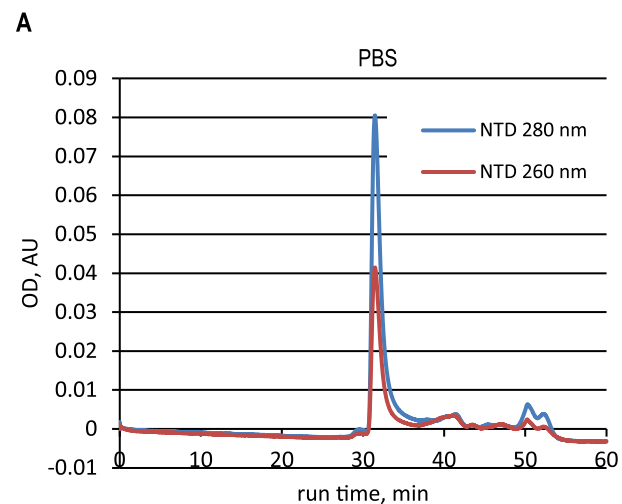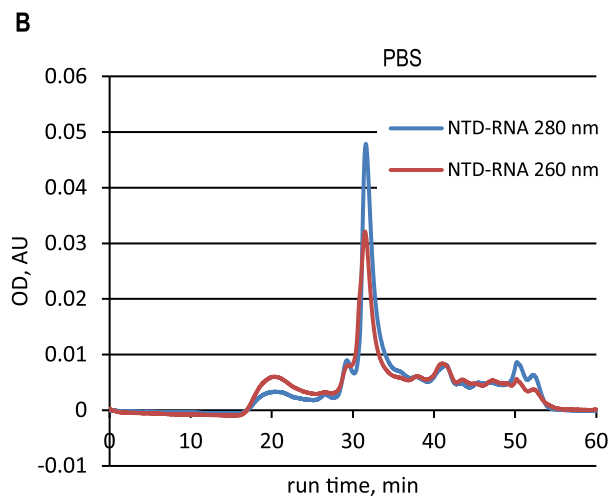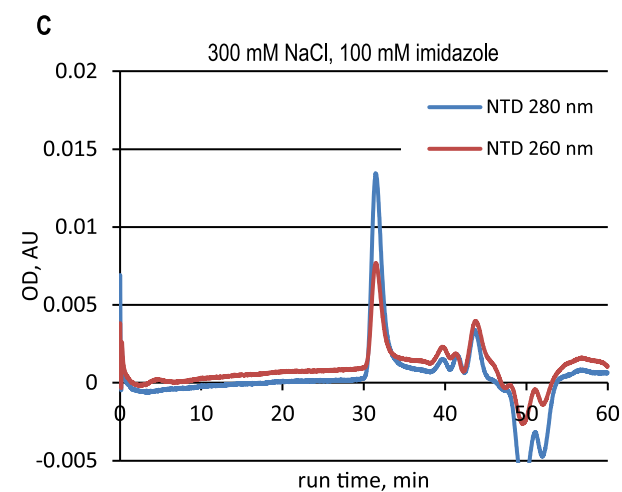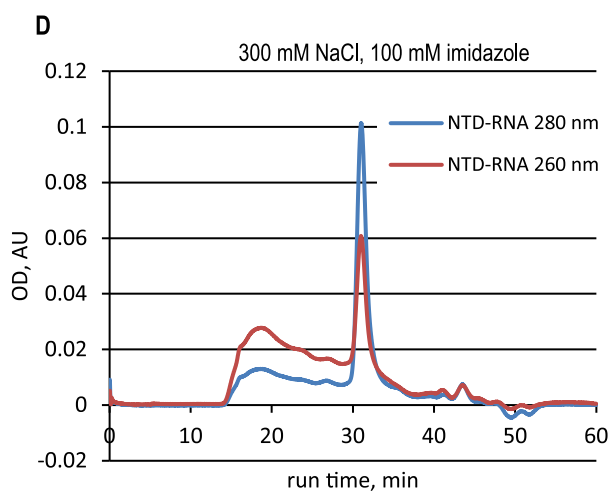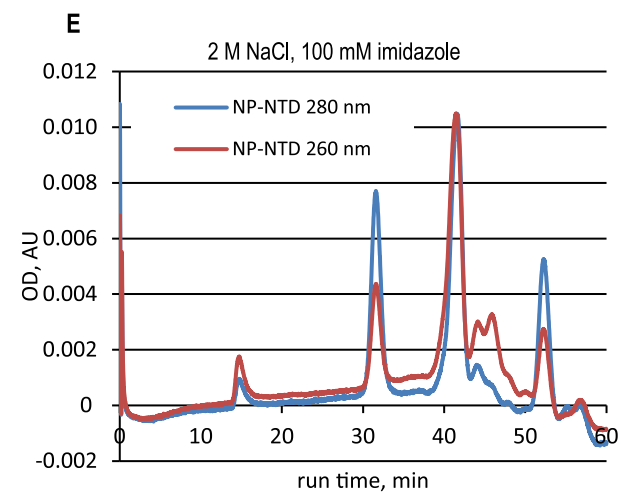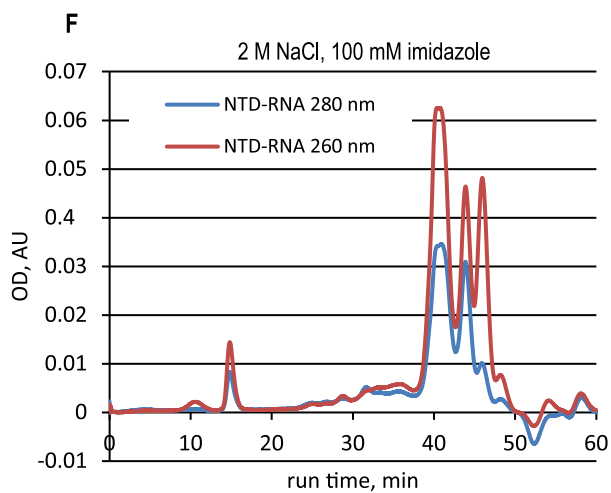

Supplement: Supplemental Information 11 — (A, B) PBS as the mobile phase; (C, D) 300 mM NaCl, 20 mM sodium phosphate pH 7.5, 100 mM imidazole-HCl mobile phase; (E, F) - 2 M NaCl, 20 mM sodium phosphate pH 7.5, 100 mM imidazole as the mobile phase. [file peerj-10-12751-s011.pdf]

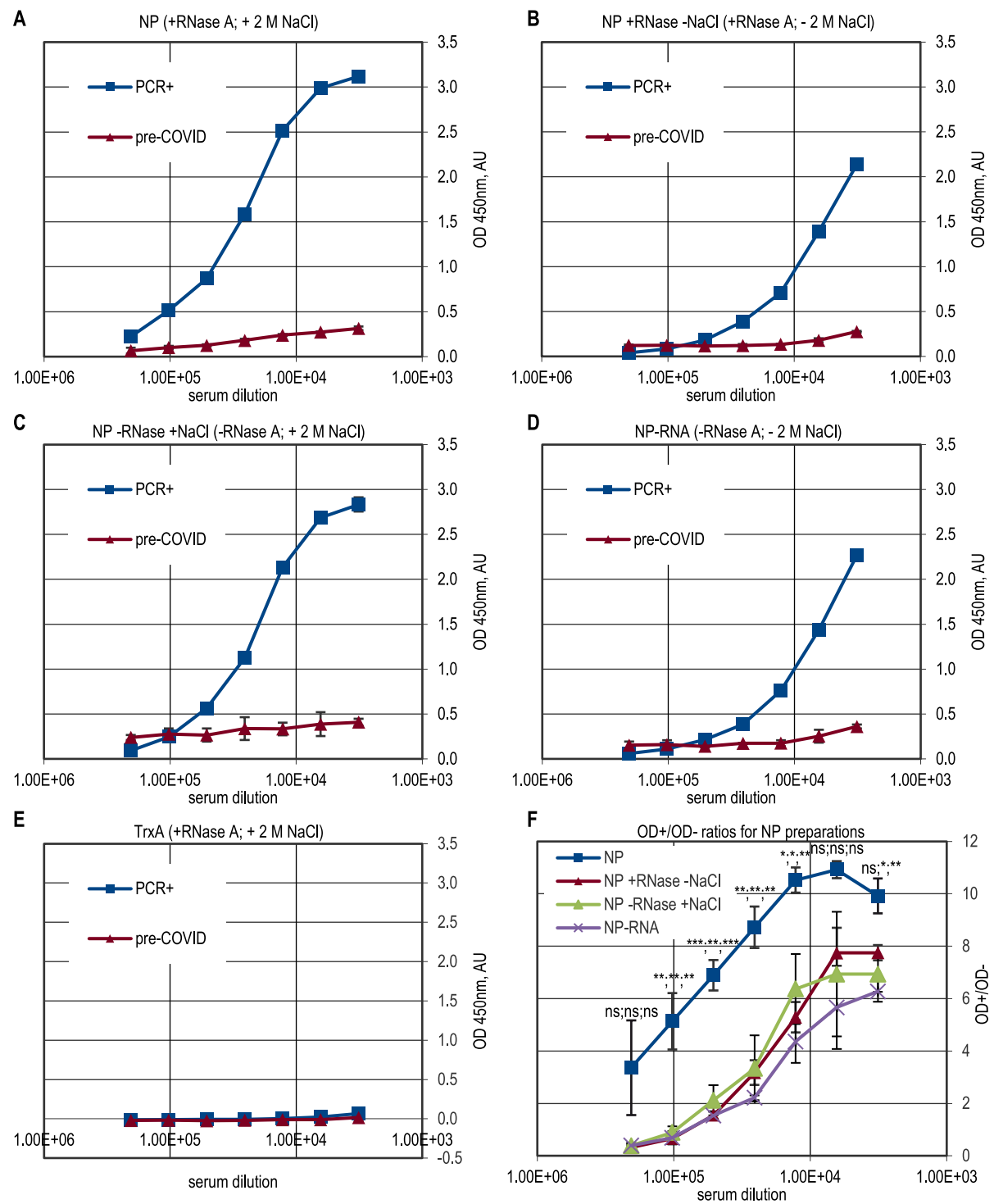

Supplement: Supplemental Information 12 — (A) pure NP antigen, protein preparation and the ELISA test was independent from the test shown on Fig. 3. (B) NP protein antigen, treated by the RNase A and not treated by the on-column 2 M NaCl wash. (C) NP protein antigen, not treated by the RNase A and treated by the on-column 2 M NaCl wash. (D) NP-RNA antigen, protein preparation and the ELISA test was independent from the test shown on Fig. 3. (E) negative control TrxA antigen, purified exactly as the NP antigen. (F) (OD+/OD-) for various serum samples dilutions, calculated as the ratios of OD readings for the PCR+ sample and the pre-COVID sample for the same sample dilutions. Blue lines - pooled PCR+ sera. Red lines - pooled pre-COVID sera. Statistical analysis by the or one-way ANOVA with the post-hoc Tukey-Kramer HSD test, n = 2, * - p < 0.05; ** - p < 0.01; *** - p < 0.001, ns–non-significant. P-values are presented for the pure NP antigen versus three other antigen preparations as “NP vs. NP+-; NP vs. NP-+; NP vs. NP-RNA”. [file peerj-10-12751-s012.pdf]

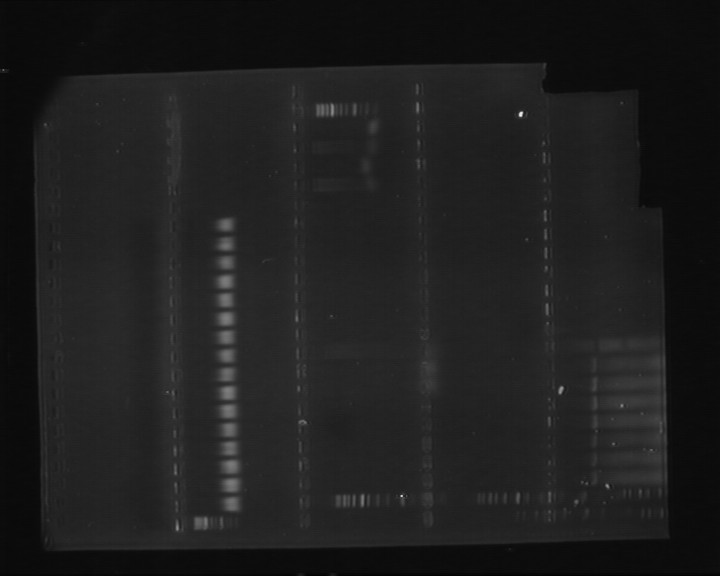

Supplement: Supplemental Information 14 — As acquired by the flatbed scanner or video camera. [file peerj-10-12751-s014.zip › NP_Fig_1_F.bmp]

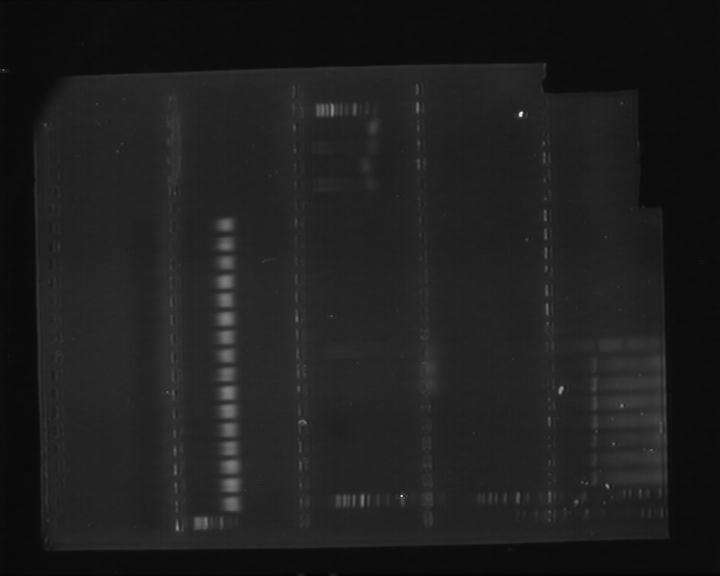

Supplement: Supplemental Information 14 — As acquired by the flatbed scanner or video camera. [file peerj-10-12751-s014.zip › NP_Fig_1_F.tif]

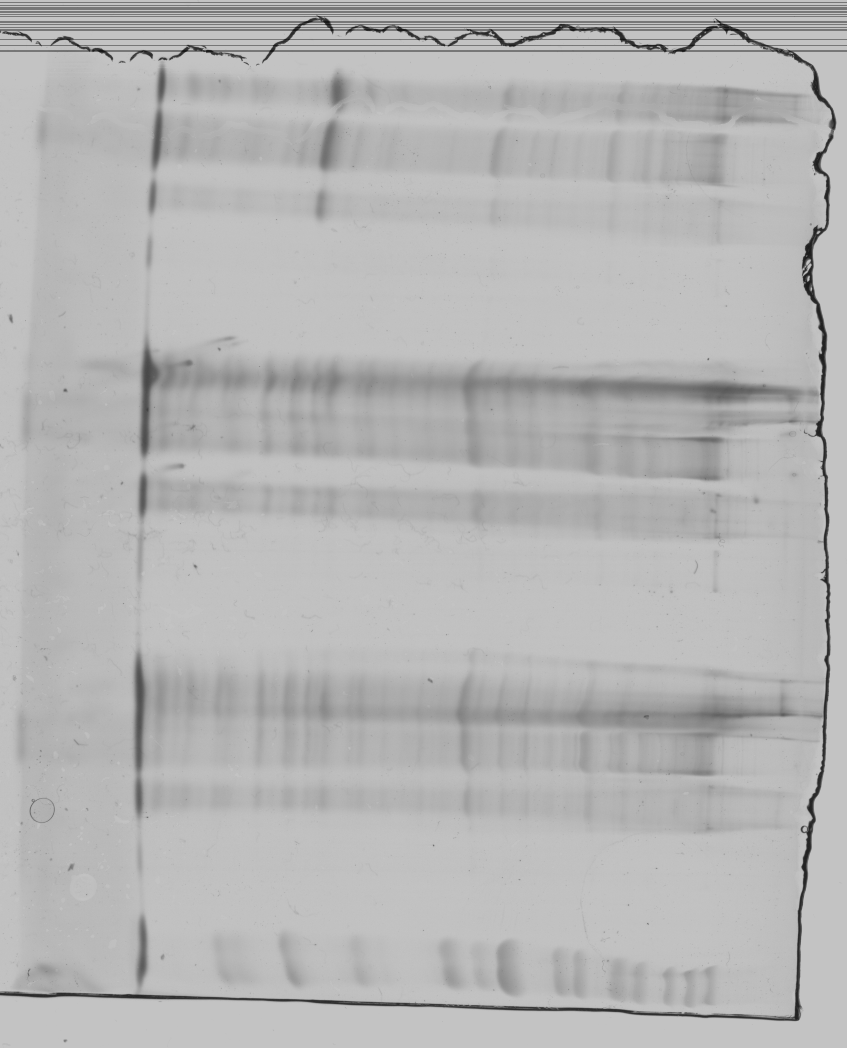

Supplement: Supplemental Information 14 — As acquired by the flatbed scanner or video camera. [file peerj-10-12751-s014.zip › NP_Fig_2_A.tif]

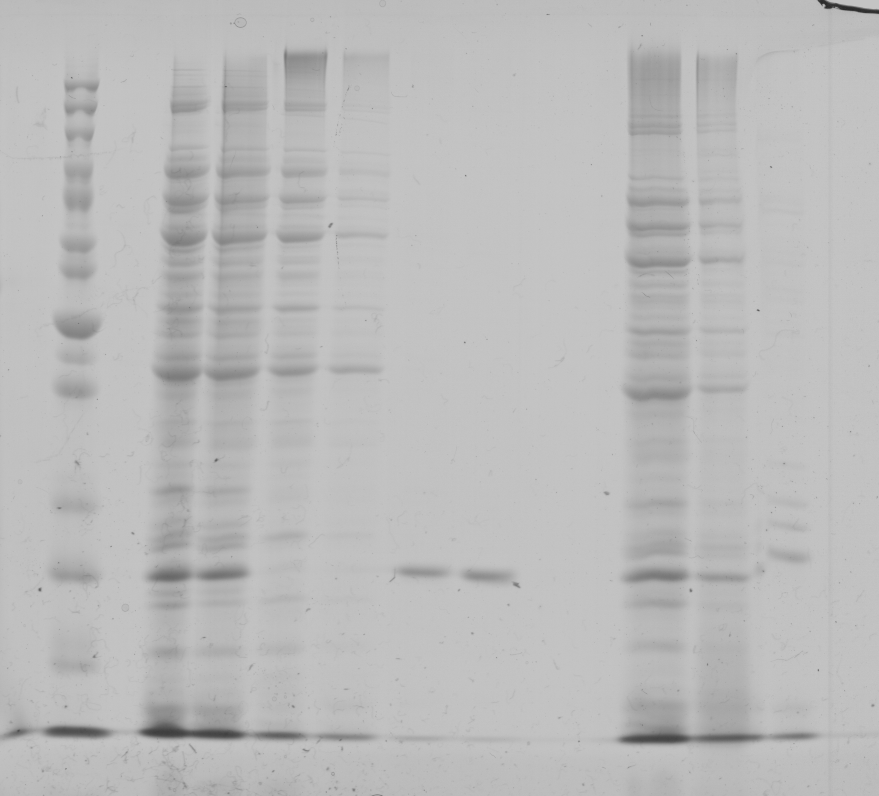

Supplement: Supplemental Information 14 — As acquired by the flatbed scanner or video camera. [file peerj-10-12751-s014.zip › NP_Fig_2_B.tif]

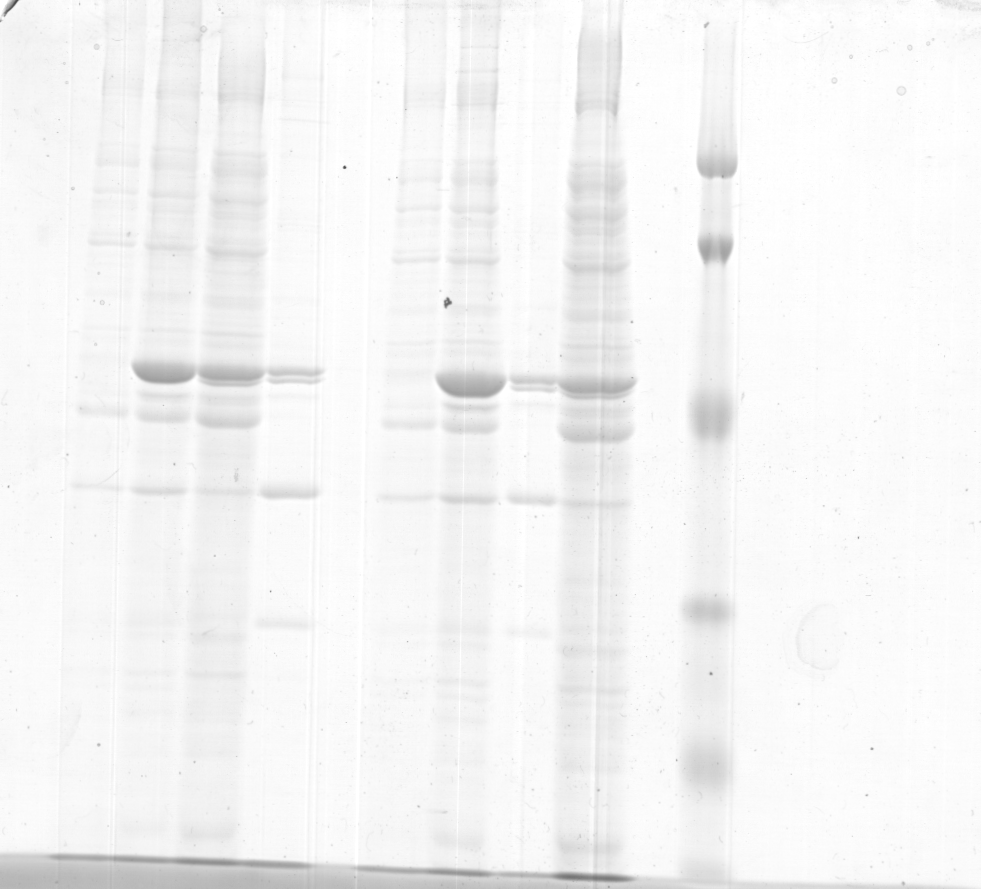

Supplement: Supplemental Information 14 — As acquired by the flatbed scanner or video camera. [file peerj-10-12751-s014.zip › NP_Fig_1_B.tif]

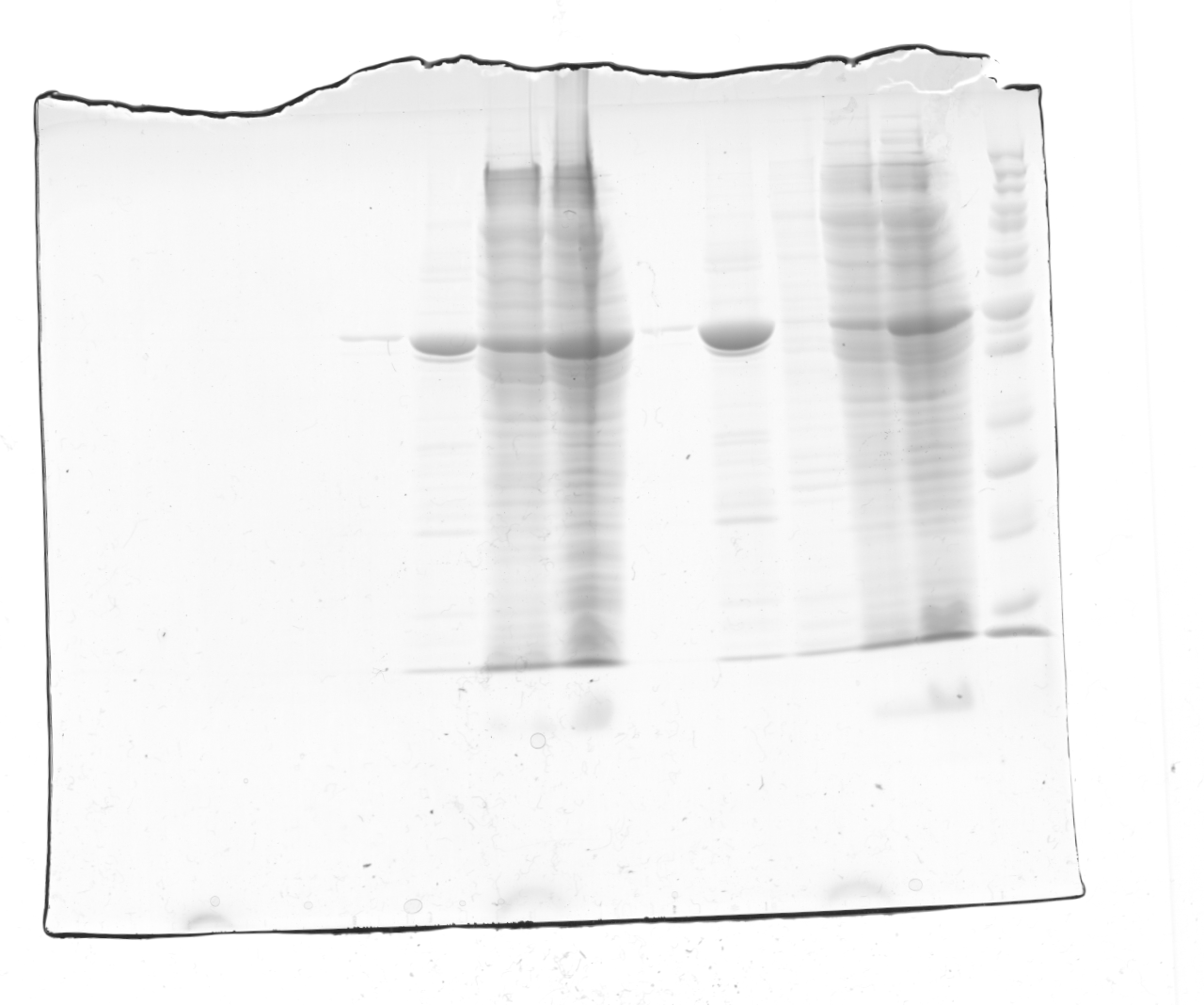

Supplement: Supplemental Information 14 — As acquired by the flatbed scanner or video camera. [file peerj-10-12751-s014.zip › NP_Fig_1_C.tif]
