# Supplementary material for: Antigenic properties of the SARS-CoV-2 nucleoprotein are altered by the RNA admixture
Source: PeerJ. 2022 Jan 7;10:e12751. doi: 10.7717/peerj.12751 (PMC8744485; doi:10.7717/peerj.12751)
Supplement: Supplemental Information 4 — SDS-PAGE analysis of total E.coli proteins, 2 h induction by 1 mM IPTG, 30 oC, total proteins (induced), soluble and insoluble protein fractions. Cl.5, cl. 7–transformation of the BL21[DE3] cells by two various clones of the pHYP-NP-CTD plasmid. [file peerj-10-12751-s004.pdf]

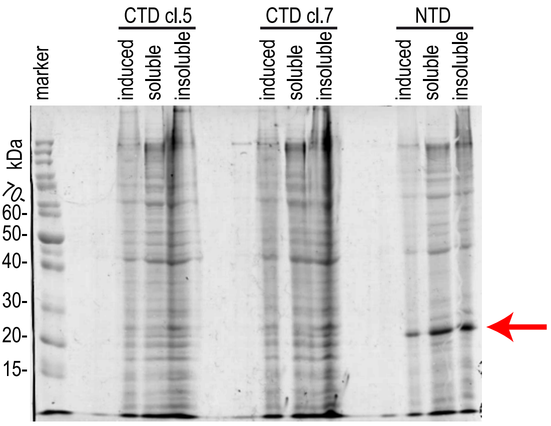
CTD cl.5
CTD cl.7
NTD
marker
induced
soluble
insoluble
induced
soluble
insoluble
induced
soluble
insoluble
kDa
70
60-
50-
40-
30-
20-
15-
