# Supplementary material for: Antigenic properties of the SARS-CoV-2 nucleoprotein are altered by the RNA admixture
Source: PeerJ. 2022 Jan 7;10:e12751. doi: 10.7717/peerj.12751 (PMC8744485; doi:10.7717/peerj.12751)
Supplement: Supplemental Information 13 — (A) pure NTD antigen, protein preparation and the ELISA test was independent from the test shown on Fig. 3. (B) NTD protein antigen, treated by the RNase A and not treated by the on-column 2 M NaCl wash. (C) NTD protein antigen, not treated by the RNase A and treated by the on-column 2 M NaCl wash. (D) NTD-RNA antigen. (E) test specificity ratios (OD+/OD-) for various serum samples dilutions, calculated as the ratios of OD readings for the PCR+ sample and the pre-COVID sample at the same sample dilutions. Blue lines - pooled PCR+ sera. Red lines - pooled pre-COVID sera. Statistical analysis by the or one-way ANOVA with the post-hoc Tukey-Kramer HSD test, n = 2, * - p < 0.05; ** - p < 0.01; *** - p < 0.001, ns–non-significant. P-values are presented for the pure NP antigen versus three other antigen preparations as “NTD vs. NTD+-; NTD vs. NTD-+; NTD vs. NTD-RNA”. [file peerj-10-12751-s013.pdf]

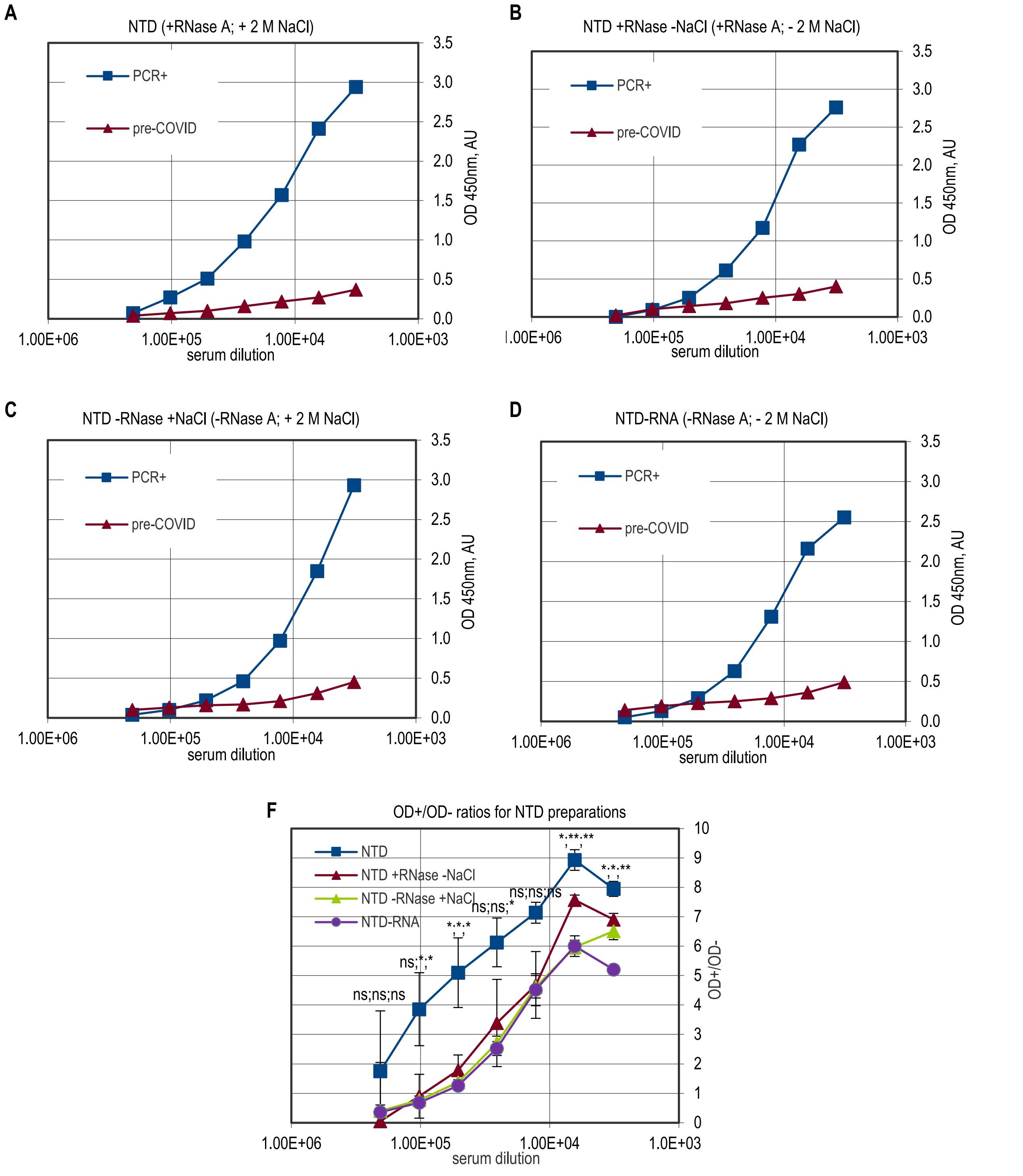

A
NTD (+RNase A; + 2 M NaCl)
PCR+
pre-COVID
OD 450nm, AU
serum dilution
B
NTD +RNase -NaCl (+RNase A; - 2 M NaCl)
PCR+
pre-COVID
OD 450nm, AU
serum dilution
C
NTD -RNase +NaCl (-RNase A; + 2 M NaCl)
PCR+
pre-COVID
OD 450nm, AU
serum dilution
D
NTD-RNA (-RNase A; - 2 M NaCl)
PCR+
pre-COVID
OD 450nm, AU
serum dilution
1.00E+06
1.00E+05
1.00E+04
1.00E+03
F
OD+/OD- ratios for NTD preparations
NTD
NTD +RNase -NaCl
NTD -RNase +NaCl
NTD-RNA
ns;ns;ns
ns;*;*
ns;ns;*
ns;ns;ns
OD+/OD-
serum dilution
1.00E+06
1.00E+05
1.00E+04
1.0E+03
